# Metataxonomic Profiling of Native and Starter Microbiota During Ripening of Gouda Cheese Made With *Listeria monocytogenes*-Contaminated Unpasteurized Milk

**DOI:** 10.3389/fmicb.2021.642789

**Published:** 2021-03-12

**Authors:** Joelle K. Salazar, Lauren J. Gonsalves, Megan Fay, Padmini Ramachandran, Kristin M. Schill, Mary Lou Tortorello

**Affiliations:** ^1^Division of Food Processing Science and Technology, Office of Food Safety, U. S. Food and Drug Administration, Bedford Park, IL, United States; ^2^Division of Microbiology, Office of Food Safety, U. S. Food and Drug Administration, College Park, MD, United States

**Keywords:** microbiome, Gouda cheese, *Listeria monocytogenes*, unpasteurized milk, dairy

## Abstract

Unpasteurized milk is used to produce aged artisanal cheeses, which presents a safety concern due to possible contamination with foodborne pathogens, especially *Listeria monocytogenes*. The objective of this study was to examine the composition of the bacterial community in unpasteurized milk used to prepare Gouda cheese artificially contaminated with *L. monocytogenes* (~1 log CFU/ml) and assess the community dynamics and their potential interaction with *L. monocytogenes* during a 90-day ripening period using targeted 16S rRNA sequencing. The diversity of bacterial taxa in three batches of unpasteurized milk was not significantly different, and the microbiomes were dominated by species of *Lactococcus*, *Streptomyces*, *Staphylococcus*, and *Pseudomonas*. The highest relative abundances were observed for *Pseudomonas fluorescens* (31.84–78.80%) and unidentified operational taxonomic units (OTUs) of *Pseudomonas* (7.56–45.27%). After manufacture, both with and without *L. monocytogenes*-contaminated unpasteurized milk, Gouda cheese was dominated by starter culture bacteria (including *Lactococcus lactis* subsp. *cremoris*, *lactis*, *lactis* bv. diacetylactis, and *Streptococcus thermophilus*), in addition to unassigned members in the taxa *L. lactis* and *Streptococcus*. During ripening there was an overall decrease in *L. lactis* abundance and an increase in the number of taxa with relative abundances >0.1%. After 90-day ripening, a total of 82 and 81 taxa were identified in the Gouda cheese with and without *L. monocytogenes*, respectively. Of the identified taxa after ripening, 31 (Gouda cheese with *L. monocytogenes*) and 56 (Gouda cheese without *L. monocytogenes*) taxa had relative abundances >0.1%; 31 were shared between the two types of Gouda cheese, and 25 were unique to the Gouda cheese without added *L. monocytogenes*. No unique taxa were identified in the Gouda cheese with the added *L. monocytogenes*. This study provides information on the dynamics of the bacterial community in Gouda cheese during ripening, both with and without the addition of *L. monocytogenes*.

## Introduction

Microbes and cheesemaking have been intertwined for hundreds of years, with the first description of cheese microorganisms dating back to 1665 (Donnelly, 2014). Many cheeses that are still consumed today originated hundreds of years ago: Cheddar, Parmesan, and Gouda and Gloucester, for example, were first documented in 1500, 1579, and 1697, respectively. Cheese serves as a long-standing dietary component in many countries around the world. Since the introduction of cheese microorganisms to science, studies have continued to shed light on the identities and roles of the organisms traditionally used in cheesemaking. The knowledge gained from these studies has resulted in a more controlled, standardized, and safe process for producing many of these cheese types (Donnelly, 2014).

Cheeses obtain their unique flavors from defined starter cultures and aging (ripening) conditions in an industrial setting that yields a predictable and replicable result. Artisanal cheesemaking, i.e., small batch, specialty-made, region-specific cheeses, relies on the selection and use of indigenous microorganism in the milk used as the base for the cheese. Supporters of artisanal cheeses argue that the high taxonomic diversity of the native microbiota in unpasteurized milk is important for developing the flavor profile unique to these cheeses. Additionally, it is debated that the diverse microbial profile of these cheeses presents competition and limits foodborne pathogens. Unfortunately, current data contradict this belief: *Listeria monocytogenes* and Shiga-toxin producing *Escherichia coli* have been identified in both unpasteurized milk and cheeses prepared using unpasteurized milk (U. S. FDA, 2016). In several studies, *L. monocytogenes* was identified in a substantial portion of the silos and bulk tanks used to house unpasteurized milk, ranging from 2.3 to 50% of the samples examined (Van Kessel et al., 2004; Jayarao et al., 2006; D’Amico et al., 2008; Jackson et al., 2012). The resident bacteria of unpasteurized milk originate from a variety of sources, including the animal’s teat canal and skin, equipment and personnel hygiene, transport and storage, and processing conditions (Verdier-Metz et al., 2009; Braem et al., 2012; Quigley et al., 2013; Kergourlay et al., 2015). Without pasteurization and proper process and post-process handling, the incidence of *L. monocytogenes* contamination may be increased. Pathogenic *E. coli* contamination of Gouda cheese and/or the unpasteurized milk that was used for manufacture has led to multiple outbreaks between 1998 and 2011 (Gould et al., 2014), and in 2013, it was responsible for 23 illnesses, five hospitalizations, and one death (Currie et al., 2018).

As the potential of foodborne illnesses remains a threat, and the consumption of artisanal cheeses increases, the interstate commerce of unpasteurized milk and cheese made with unpasteurized milk remains closely monitored and is illegal except under certain guidelines. Under U.S. Food and Drug Administration (FDA) regulations in the Code of Federal Regulations (CFR) Title 21 (U. S. FDA, 2018), specific standards allow cheese to be made from unpasteurized milk, but must be cured (aged) at a temperature not lower than 1.7°C (35°F) and for a minimum of 60 days, in an effort to reduce pathogen presence. However, multiple studies have indicated that in aged cheese, such as Cheddar and Gouda, the presence of a pathogen can extend well beyond the recommended 60-day ripening period (Reitsma and Henning, 1996; Schlesser et al., 2006; Salazar et al., 2020).

The demand for high-quality artisanal and traditional cheeses likely will not diminish, nor will the incidence of foodborne pathogens in these products. Little knowledge exists on how the resident microbiota impacts pathogen survival and whether altering specific taxa can aid in pathogen prevention measures in the artisanal cheese industry. The present study, complementing previous research (Salazar et al., 2020), assesses the microbial composition of Gouda cheese made with unpasteurized milk during a 90-day ripening process. The goals of this study were to determine the change in abundance of the taxa present (both from the starter culture, as well as those naturally residing in the milk) and to observe dynamic changes in the presence of *L. monocytogenes*, in an effort to provide information that may prove valuable for the development of future guidelines and risk assessments.

## Materials and Methods

### Strains and Inoculum Preparation

A cocktail of four *L. monocytogenes* dairy-related strains were used in this study: LM1240, LM1257, LM-6E, and LM-2F (van der Veen et al., 2008; Wemmenhove et al., 2013). Strains were cultured individually aerobically in Brain Heart Infusion (BHI; Becton, Dickinson and Co., Sparks, MD) broth at 37°C for 16–18 h. Cultures were normalized to an OD_600_ of 0.8, washed twice with phosphate-buffered saline (PBS; pH 7.4), and combined to form a cocktail. The cocktail was serially diluted using PBS and plated onto Brilliance *Listeria* agar (BLA; Thermo Fisher Scientific, Waltham, MA) to verify initial inoculum levels.

### Gouda Cheese Manufacture

Three batches of unpasteurized bovine milk (AB, CD, and EF) were sourced from a local dairy in Illinois on different concurrent weeks. Each batch of unpasteurized milk received was comprised of milk from multiple farms in Illinois. A total of three trials and six Gouda cheeses were prepared (labeled A through F). Trial 1 used batch AB unpasteurized milk to manufacture control uninoculated cheese A and *L. monocytogenes*-contaminated cheese B. Trial 2 used batch CD unpasteurized milk to manufacture control uninoculated cheese C and *L. monocytogenes*-contaminated cheese D. Trial 3 used batch EF unpasteurized milk to manufacture control uninoculated cheese E and *L. monocytogenes*-contaminated cheese F.

Gouda cheese was manufactured in a biocontainment pilot plant using a commercial cheese pasteurization vat (V15005, Northwestern Tools, Dayton, OH) as previously described (Salazar et al., 2020). Briefly, 10 gallons (37.9 L) of unpasteurized milk was added to the vat and was left uninoculated (in the case of cheeses A, C, and E) or was inoculated with 1.05 ± 0.24 log CFU/ml of the *L. monocytogenes* cocktail (in the case of cheeses B, D, and F). The milk was then heated to 30°C, followed by the addition of 2.4 Direct Culture Unit (DCU) of starter cultures consisting of *Lactococcus lactis* subsp. *lactis*, *L. lactis* subsp. *cremoris*, *L. lactis* subsp. *lactis* bv. diacetylactis, and *Streptococcus thermophilus* (CHOOZIT MA 4001 Lyo 25 DCU, Danisco, Thomson, IL). After 30 min at 30°C, 6.2 ml of rennet (PF 55 coagulant, GetCulture, Madison, WI) was added to the milk, mixed, and the temperature was maintained for 45–55 min. Curds were cut in up and down directions with a 1 inch^2^ cheese knife, with a 5 min wait between cuts. One third of the whey was drained, followed by the addition of an equal amount of water at 50°C, which aided in increasing the overall temperature of the mixture to 38°C. The curds were “cooked” at 38°C for 30 min with constant stirring. Curd were placed into molds (M19, New England Cheesemaking Supply Co., Deerfield, MA) and pressed with sequential increasing weights as described previously (Salazar et al., 2020). The resulting cheese wheel was brined in 20% (w/v) sodium chloride (Thermo Fisher Scientific) for 48 h, followed by 72 h ambient drying prior to waxing.

### Waxing, Storage, and Sampling of Gouda Cheese During Ripening

Gouda cheese was waxed, stored, and sampled as described previously (Salazar et al., 2020). Cheese wheels were waxed with two coats of red cheese wax (New England Cheesemaking Supply Co.) and aged at 10°C for up to 90 days. At 0, 7, 28, 42, 60, 77, and 90 days, a 25-g pie-shaped wedge of cheese was removed from the wheel for sampling. The cheese was re-waxed after each sampling with two coats of cheese wax. All Gouda cheeses prepared in this study conformed to the standard of identity of Gouda cheese (U. S. FDA, 2018; Salazar et al., 2020).

### Sample Processing and Sequencing

DNA was extracted from 1 ml of each of the three batches of unpasteurized milk (AB, CD, and EF) prior to the manufacture of the Gouda cheese to determine the composition and relative abundance of bacterial taxa. After the Gouda cheese was prepared, individual 25-g cheese samples, consisting of two 5-g pie-shaped wedges were evaluated. Each 5-g sample was homogenized with 10 ml of Buffered *Listeria* Enrichment Broth (BLEB; Oxoid, Basingstoke, England), and DNA was extracted immediately from 1 ml of each homogenate using the DNeasy PowerFood Microbial Kit (Qiagen, Hilden, Germany) according to the manufacturer’s instructions. Total DNA was quantified using the Qubit dsDNA BR Assay Kit (Invitrogen, Carlsbad, CA). PCR was conducted with 3 ng of template DNA and one of four 16S rDNA primer pairs to amplify the V4 region as previously described (Salazar et al., 2018). Amplicons were purified using AMPure XP beads (Beckman-Coulter, Indianapolis, IN) and quantified using the Qubit dsDNA BR Assay Kit. Samples were indexed using the Nextera XT Kit (Illumina, San Diego, CA) as described previously (Salazar et al., 2018), pooled, spiked with 10% of 12.5 pM PhiX, and sequenced using an Illumina MiSeq and 600 cycles of V3 chemistry.

### Data Analyses

Raw paired-end sequences were quality filtered, merged, and taxonomically profiled using miniKraken and Kraken2 (Wood et al., 2019). Relative abundances were determined using Bracken 2.5 (Lu et al., 2017). Sequence counts were rarefied to 10,000 sequences for each independent sample. NCBI BLAST+ 2.9.0 was used to differentiate between the *L. lactis* subspecies (i.e., *lactis*, *cremoris*, and *lactis* bv. diacetylactis) using the BLAST V5 database. The vegan package 2.5–6 (Oksanen et al., 2019) in R 3.6.2 was used to determine alpha and beta diversity of the microbial population in the three unpasteurized milk batches (AB, CD, and EF), the six Gouda cheeses (A, B, C, D, E, and F), and the two types of cheeses (ACE, uninoculated; BDF, inoculated). Alpha diversity was estimated using Shannon, Simpson, inverse Simpson, and Chao1 diversity indices. Beta diversity was evaluated using Bray-Curtis dissimilarity matrix. A multilevel pairwise comparison using Adonis (~Permanova) was calculated using package pairwiseAdonis (Martinez Arbizu, 2020). A value of *p* less than 0.05 was considered as significant.

### Accession Numbers

Metagenomic sequence data have been deposited in NCBI under Bioproject PRJNA643290, Biosamples SAMN15409189-409233.

## Results

### Native Bacteria in the Unpasteurized Milk

**Figure 1:**
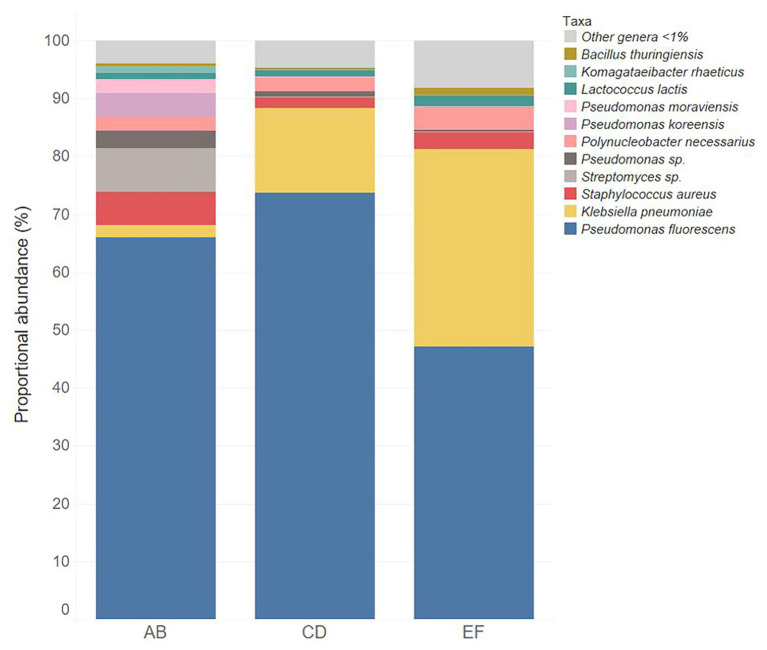
Relative abundance of bacterial taxa in the three batches of unpasteurized milk (AB, CD, and EF) used in this study used to make Gouda cheese.

**Figure 2:**
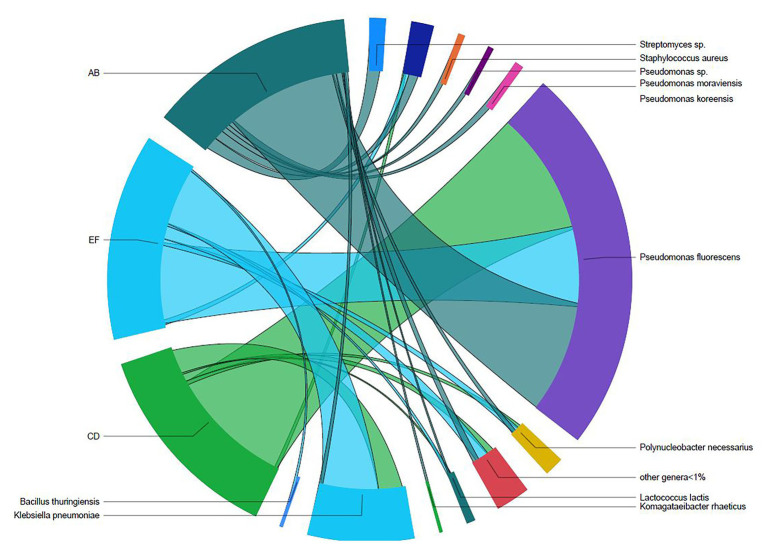
Shared taxa between the three batches of unpasteurized milk (AB, CD, and EF) used to manufacture Gouda cheese.

Three batches of unpasteurized milk were used to manufacture the six Gouda cheeses in this study. From plate count assays conducted previously (Salazar et al., 2020), the average populations of *Enterobacteriaceae*, yeasts and molds, lactic acid bacteria, and mesophilic bacteria in the unpasteurized milk were 0.95, 3.53, 3.33, and 3.09 log CFU/ml, respectively. In this study, a total of 193, 206, and 176 unique operational taxonomic units (OTUs) were identified in unpasteurized milk batch AB, CD, and EF, respectively (Table 1). Although differences were observed in the total OTUs, alpha and beta diversity metrics were not significantly different between batches (Tables 1 and 2). The bacterial genera *Lactococcus*, *Pseudomonas*, *Polynucleobacter*, *Streptomyces*, and *Staphylococcus* dominated the milk microbiomes (Figures 1, 2). A total of 11 different taxa were identified with relative abundances >1% in at least one of the three milk batches and included *L. lactis*, *Pseudomonas fluorescens*, *P. koreensis*, *P. moraviensis*, *Polynucleobacter necessarius*, *Staphylococcus aureus*, *Bacillus thuringiensis*, *Klebsiella pneumoniae*, and *Komagataeibacter rhaeticus*, as well as unclassified members in the genera of *Streptomyces* and *Pseudomonas*. The highest relative abundance was observed for *P. fluorescens* (47.06–73.76%). *Lactococcus lactis* was present in all three milk batches at relative abundances of 1.03–1.81%. Differences in the relative abundances of some taxa were more pronounced than others in the three batches of milk (Figures 1, 2). *Klebsiella pneumoniae* was present in all three milk batches but at different relative abundances (2.24–34.17%). A high abundance of *Streptomyces* (7.60%) was observed in milk batch AB, whereas this taxon had a relative abundance <1% in the other two batches. *Staphylococcus aureus* was also present in all three milk batches at 2.03–5.60%. No native *Listeria* spp. were detected in the unpasteurized milk *via* 16S rRNA sequencing. In addition, no native *Listeria* spp. were detected in the unpasteurized milk through enrichments (Salazar et al., 2020).

**Table 1:** Observed operational taxonomic units (OTUs) and alpha diversity metrics of the three batches of unpasteurized milk (AB, CD, and EF) used for Gouda cheese manufacture.

Milk batch	Observed OTUs	Chao1^a^	Shannon^b^	Simpson^c^	Inverse Simpson^c^
AB	193 ± 51	465 ± 89	1.35 ± 0.11	0.64 ± 0.04	2.89 ± 0.55
CD	206 ± 72	449 ± 52	1.02 ± 0.04	0.55 ± 0.01	2.23 ± 0.04
EF	176 ± 43	486 ± 96	1.04 ± 0.27	0.36 ± 0.24	1.62 ± 0.95

a Chao1 diversity index indicates the microbial richness for the three different batches of unpasteurized milk.

b Shannon diversity index indicates the richness and evenness of the microbial community in the three different batches of unpasteurized milk.

c Simpson and Inverse Simpson diversity indices indicate the diversity of the microbial community in the three batches of unpasteurized milk.

**Table 2:** Pairwise Adonis of the three batches of unpasteurized milk used for Gouda cheese manufacture.

Comparison	Sums of Squares^a^	F.Model^b^	*r*^2^^c^	*p*	Adjusted value of *p*^d^
AB vs. CD	0.41	1.56	0.24	0.07	0.21
AB vs. EF	0.38	1.31	0.25	0.133	0.4
CD vs. EF	0.32	1.28	0.30	0.1	0.3

a Sums of Squares expresses the total variation.

b F.Model expresses the variation between the two unpasteurized milk batches compared.

c *r*^2^, coefficient of determination.

d Adjusted value of *p*, the value of *p* obtained using Bonferroni correction.

### Native and Starter Bacteria in Manufactured Gouda Cheese Prior to Ripening

**Figure 3:**
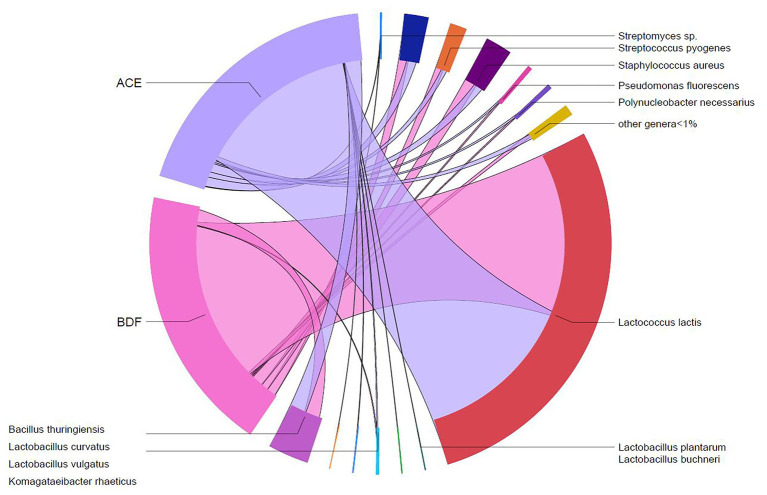
Shared taxa between the two types of Gouda cheeses (ACE, uninoculated and BDF, inoculated) produced in this study.

**Figure 4:**
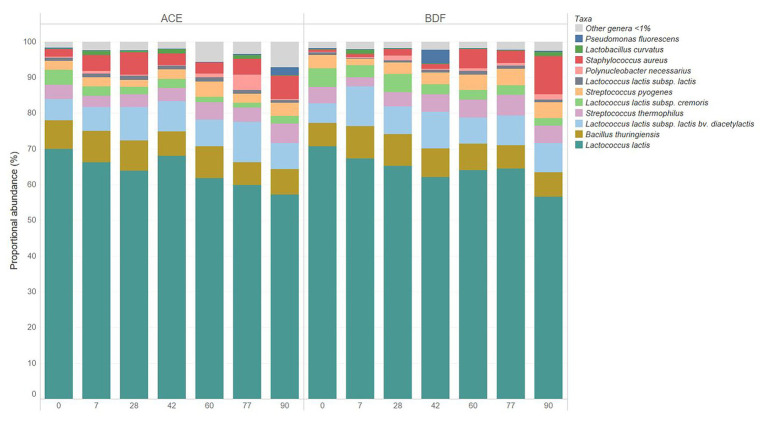
Relative abundance of bacterial taxa during 10°C aging of Gouda cheese manufactured with unpasteurized milk (cheese ACE) or with unpasteurized milk inoculated with 1 log CFU/mL of *Listeria monocytogenes* (cheese BDF).

Two types of Gouda cheese were manufactured in this study: one type was manufactured with unpasteurized milk without addition of *L. monocytogenes* (cheese ACE) and one manufactured with unpasteurized milk containing 1.05 ± 0.24 log CFU/ml of *L. monocytogenes* (cheese BDF). From plate count assays conducted previously (Salazar et al., 2020), the average populations of *Enterobacteriaceae*, yeasts and molds, lactic acid bacteria, and mesophilic bacteria in the manufactured cheeses prior to ripening were 2.07, 3.06, 8.98, and 8.83 log CFU/g, respectively. Relative abundances of native and starter bacteria in the two types of Gouda cheeses (ACE and BDF) were determined at day 0 (after manufacture) and during ripening at 10°C for up to 90 days. At day 0, a total of 85 different taxa were identified in cheese ACE. Similarly, 85 different taxa were also identified in cheese BDF. Excluding starter culture bacteria, only two (*B. thuringiensis* and *P. fluorescens*) of the 11 taxa identified in the unpasteurized milk >1% (Figure 1) were present in both types of Gouda cheese at relative abundances >1% (Figures 3, 4). *Bacillus thuringiensis* was present in cheese ACE and cheese BDF at relative abundances of 7.97 and 6.58%, respectively. *Pseudomonas fluorescens*, which was identified in the unpasteurized milk at 47.06–73.76%, had reduced relative abundances of 0.23 (cheese ACE) and 0.26% (cheese BDF). *Staphylococcus aureus* was present in cheese ACE at a relative abundance of 2.12%, however, it was <1% in cheese BDF.

The starter culture used for Gouda cheese manufacture consisted of four different bacteria: *L. lactis* subsp. *lactis*, *L. lactis* subsp. *cremoris*, *L. lactis* subsp. *lactis* bv. diacetylactis, and *S. thermophilus*. No differences in relative abundances of the starter culture bacteria were observed between the two types of Gouda cheeses (ACE and BDF) on day 0 (Figures 3, 4). *Lactococcus lactis* subsp. *lactis* bv. diacetylactis was present at an average relative abundance of 5.73%, followed by *L. lactis* subsp. *cremoris* and subsp. *lactis* at 4.68 (cheese ACE) and 0.78% (cheese BDF). A high abundance of *L. lactis* that was not categorized into subtypes was present in both types of Gouda cheeses at an average abundance of 70.32%. *Streptococcus thermophilus*, another starter culture bacterium, was identified at an average relative abundance of 4.28%.

### Influence of *Listeria monocytogenes*-Contaminated Milk on the Gouda Cheese Microbiome During Ripening

**Figure 5:**
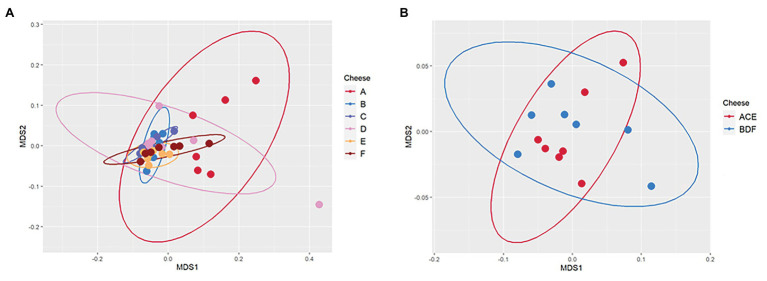
Principal Component Analysis Plot (PCA) using Bray Curtis distance matrix to measure beta diversity of **(A)** the six individual Gouda cheeses (A, B, C, D, E, and F) and **(B)** the two types of Gouda cheeses (ACE, uninoculated and BDF, inoculated) manufactured in this study.

During 90-day ripening, beta diversity analysis determined that there were significant differences between the microbiomes of cheeses A and C as well as A and E, all of which were uninoculated (Table 3 and Figure 5). No significant differences were observed between the microbiomes of any of the three inoculated cheeses (B, D, and F). In addition, there was a significant difference between the microbiomes of the two types of cheeses manufactured in this study (ACE and BDF; Table 3 and Figure 5).

**Table 3:** Pairwise Adonis of the six individual Gouda cheeses (A, B, C, D, E, and F) as well as the two types of cheeses (ACE, uninoculated and BDF, inoculated) manufactured in this study.

Comparison	Sums of Squares^a^	F.Model^b^	*r*^2^^c^	*p*	Adjusted value of *p*^d^
A vs. B	0.15	2.48	0.17	0.02	0.3
A vs. C	0.30	6.23	0.34	0.001	0.015^*^
A vs. D	0.19	3.47	0.22	0.008	0.12
A vs. E	0.29	5.98	0.32	0.002	0.03^*^
A vs. F	0.23	4.98	0.29	0.004	0.06
B vs. C	0.07	1.94	0.14	0.072	1
B vs. D	0.05	1.10	0.08	0.371	1
B vs. E	0.14	3.47	0.21	0.004	0.06
B vs. F	0.07	1.99	0.14	0.071	1
C vs. D	0.05	1.65	0.12	0.128	1
C vs. E	0.06	2.29	0.15	0.033	0.495
C vs. F	0.04	1.80	0.13	0.103	1
D vs. E	0.10	3.06	0.19	0.012	0.18
D vs. F	0.04	1.32	0.10	0.025	1
E vs. F	0.03	1.00	0.07	0.402	1
ACE vs. BDF	0.07	1.96	0.14	0.042	0.042^*^

* Indicates a significant difference.

a Sums of Squares expresses the total variation.

b F.Model expresses the variation between the two cheeses compared.

c *r*^2^, coefficient of determination.

d Adjusted value of *p*, the value of *p* obtained using Bonferroni correction.

The relative abundances of native, starter, and *L. monocytogenes* were determined during the 90-day ripening of the two types of Gouda cheeses (Figures 3, 4). *L. monocytogenes* was identified at relative abundances less than 0.001% in Gouda cheese BDF during ripening, which was consistent with previously determined plate count enumeration data (Salazar et al., 2020); after 90-day ripening, the population of *L. monocytogenes* was 1.11, 0.95, and 1.26 log CFU/g in cheeses B, D, and F, respectively. *L. monocytogenes* was not identified in cheese ACE (uninoculated) at any timepoint during ripening using 16S rRNA gene sequencing or via enrichments (Salazar et al., 2020).

Non-starter culture bacteria identified during ripening at relative abundances >1% for at least one timepoint in either type of Gouda cheese included *B. thuringiensis*, *Lactobacillus curvatus*, *Po. necessarius*, *P. fluorescens*, *S. aureus*, and *Str. pyogenes* (Figures 3, 4). *Lactobacillus curvatus* was identified at relative abundances of 0.03–2.25% and <0.1–1.26% in cheeses ACE and BDF during ripening, respectively. *Polynucleobacter necessarius* was identified at relative abundances of 0.24–4.35% and 0.15–1.41% in cheeses ACE and BDF during ripening, respectively. *Bacillus thuringiensis* (6.30–9.11%) and *Str. pyogenes* (1.78–4.63%) were the only two taxa present in both ACE and BDF cheeses at all timepoints during ripening. *S. aureus* was also present in both cheeses at all timepoints and increased during ripening at relative abundances of 2.13–10.73% with the exception of 0 and 7 days for cheeses BDF in which the relative abundances were 0.81 and 0.82%, respectively. From initial relative abundances of 2.13 and 0.81% on day 0, the abundance of *S. aureus* after 90 days ripening was 6.61 and 10.73% for cheeses ACE and BDF, respectively.

Less difference was observed in the relative abundances of starter culture bacteria between the two types of cheeses during ripening (Table 4). In general, the relative abundance of *L. lactis* subsp. *lactis* remained fairly constant during ripening. The relative abundance of *L. lactis* subsp. *cremoris* was observed to decrease during ripening. Conversely, the relative abundance of *L. lactis* subsp. *lactis* bv. diacetylactis increased over the 90-day ripening period. The greatest increase in relative abundance (2.71%) was observed by *L. lactis* subsp. *lactis* bv. diacetylactis in cheese BDF, whereas the greatest decrease (14.05%) was observed by unassigned *L. lactis* members, also in cheese BDF.

**Table 4:** Average relative abundances of starter culture bacteria during 10°C ripening of Gouda cheese manufactured with unpasteurized milk (cheeses ACE) or with unpasteurized milk inoculated with 1 log CFU/ml of *Listeria monocytogenes* (cheese BDF).

Starter culture bacteria	Relative abundance (%)			
	0 day	28 days	60 days	90 days
**Cheese ACE**				
*Lactococcus lactis* subsp. *cremoris*	4.17	2.11	1.45	2.20
*Lactococcus lactis* subsp. *lactis*	0.90	1.17	1.14	0.84
*Lactococcus lactis* subsp. *lactis* bv. diacetylactis	5.97	9.23	7.54	7.29
*Lactococcus lactis* unassigned OTUs	69.97	63.83	61.78	57.19
*Streptococcus thermophilus*	3.96	3.64	4.86	5.47
**Cheese BDF**				
*Lactococcus lactis* subsp. *cremoris*	5.18	5.08	2.74	2.09
*Lactococcus lactis* subsp. *lactis*	0.66	0.69	0.99	0.82
*Lactococcus lactis* subsp. *lactis* bv. diacetylactis	5.50	7.63	7.36	8.21
*Lactococcus lactis* unassigned OTUs	70.67	65.23	64.08	56.62
*Streptococcus thermophilus*	4.59	4.05	4.97	4.92

When comparing cheeses ACE and BDF, after 28, 60, and 90 days ripening, a total of 98, 130, and 81 taxa were identified in Gouda cheese ACE, respectively, whereas 95, 77, and 82 were identified in cheeses BDF. Out of the 81 taxa identified in cheese ACE after 90 days, 25, 47, and 9 taxa had relative abundances <0.1%, 0.1–0.5, and >0.5%, respectively. In contrast, out of 82 taxa identified in cheese BDF after 90 days, 51, 26, and 5 taxa had relative abundances <0.1%, 0.1–0.5, and >0.5%. Of the taxa with relative abundances 0.1–0.5%, 26 were shared between both types of cheeses and 21 were only identified in cheese ACE; no taxa were unique to cheese BDF, with the exception of the inoculated *L. monocytogenes* (Table 5). Genera with the most unique species identifications included *Bacteroides* and *Prevotella*.

**Table 5:** Taxa present at relative abundances of 0.1–0.5% in Gouda cheese manufactured with unpasteurized milk (cheese ACE) or with unpasteurized milk inoculated with 1 log CFU/ml of *Listeria monocytogenes* (cheese BDF) after 90 days aging at 10°C.

Taxa unique to ACE	Taxa present in both ACE and BDF
*Aerococcus christensenii* *Amycolatopsis mediterranei* *Anaerotignum propionicum* *Bacteroides cellulosilyticus* *Bacteroides dorei* *Bacteroides helcogenes* *Bacteroides heparinolyticus* *Bacteroides thetaiotaomicron* *Basilea psittacipulmonis* *Flavobacterium johnsoniae* *Lactobacillus crispatus* *Limnohabitans* spp. *Macrococcus caseolyticus* *Photobacterium damselae* *Porphyromonas gingivalis* *Prevotella dentalis* *Prevotella fusca* *Prevotella intermedia* *Prevotella melaninogenica* *Prevotella ruminicola* *Prevotella scopos*	*Bacillus thuringiensis* *Bacteroides ovatus* *Denitrovibrio acetiphilus* *Faecalibacterium prausnitzii* *Fimbriimonas ginsengisoli* *Haemophilus parainfluenzae* *Ketobacter alkanivorans* *Lactiplantibacillus pentosus* *Lactiplantibacillus plantarum* *Lacticaseibacillus rhamnosus* *Levilactobacillus brevis* *Ligilactobacillus acidipiscis* *Ligilactobacillus murinus* *Limosilactobacillus fermentum* *Loigolactobacillus coryniformis* *Odoribacter splanchnicus* *Paenisporosarcina* spp. *Propionibacterium freudenreichii* *Pseudomonas koreensis* *Pseudomonas mandelii* *Sporosarcina* spp. *Streptococcus anginosus* *Streptococcus halotolerans* *Streptococcus pyogenes* *Streptomyces* spp. *Syntrophus aciditrophicus*

No unique taxa were identified in cheese BDF, with the exception of the inoculated *Listeria monocytogenes*.

## Discussion

This study used metagenomic sequencing to profile the microbiomes of unpasteurized milk and the resulting Gouda cheese after manufacture and throughout a 90-day ripening period. Although previous studies have examined the resident bacterial populations of unpasteurized cheeses after manufacture and during the ripening process (Delbès et al., 2007; Casalta et al., 2009; Lusk et al., 2012; Quigley et al., 2012; Fuka et al., 2013; Delcenserie et al., 2014; Alessandria et al., 2016; Escobar-Zepeda et al., 2016), this is the first study to assess the community dynamics both with and without the addition of a foodborne pathogen. Contamination of unpasteurized milk can occur on the farm due to poor hygiene or unsanitary conditions, or during processing. *L. monocytogenes* has been identified in unpasteurized milk in various studies (Van Kessel et al., 2004; Jayarao et al., 2006; D’Amico et al., 2008; Jackson et al., 2012). Therefore, in this study *L. monocytogenes* was added to unpasteurized milk used to produce Gouda cheese to understand the dynamics and interactions of this pathogen with the bacterial community present during ripening.

In this study, the microbiomes of the three batches of unpasteurized milk utilized for Gouda cheese manufacture had similar bacterial diversity and were dominated by *Lactococcus*, *Klebsiella*, *Pseudomonas*, *Staphylococcus*, and *Streptococcus* species. These genera have all been previously identified in unpasteurized milk (Correa et al., 2011; Quigley et al., 2013; Sudarwanto et al., 2015; Kable et al., 2016; Rodrigues et al., 2017) and *Streptococcus*, *Staphylococcus*, and *Pseudomonas* have been identified at abundances >30% in unpasteurized milk tanker trucks (Kable et al., 2016). Specifically, *P. fluorescens*, which was identified in the unpasteurized milk batches at abundances of 32–79%, is a known dairy spoilage organism and is responsible for blue discoloration in different types of cheeses (Ternstrom et al., 1993; Martin et al., 2011; del Olmo et al., 2018). These bacteria have also been shown to be inhibitory to *L. monocytogenes* in co-cultures (Buchanan and Bagi, 1999). It is noted that the unpasteurized milk batches used in this study were sourced from only one dairy; however, milk from multiple farms within the same geographic region comprised the batches. Although the microbiome of unpasteurized milk would likely vary based on the types of cattle at each farm, differences attributed to the geographic location were minimized.

After manufacture both with and without the addition of *L. monocytogenes* in the unpasteurized milk, the Gouda cheese bacterial communities were dominated by the starter culture members *L. lactis* subsp. *lactis*, *L. lactis* subsp. *cremoris*, *L. lactis* subsp. *lactis* bv. diacetylactis, and *Str. thermophilus*, as well as *L. lactis* that was not classified into subspecies and unclassified species in the genus *Streptococcus*. This finding was expected, as high population levels of starter culture bacteria are common in cheeses after manufacture (Montel et al., 2014). The strains in starter cultures play an important role in the cheese manufacturing and ripening process by influencing sensory, flavor, and texture profiles. This study utilized a commercially-available Gouda cheese starter culture. During cheese manufacture, the main contribution of these lactic acid bacteria is in milk acidification. During ripening, these bacteria are also involved in lipolysis, proteolysis, and the conversion of amino acids to flavor compounds (Yvon et al., 1997; Copan et al., 2007). Since the relative abundances of the strains in the starter cultures were similar between cheeses ACE and BDF during ripening, it can be inferred that *L. monocytogenes* is not inhibitory to the starter culture bacteria and vice versa.

After manufacture, the Gouda cheeses made with unpasteurized milk, both with and without the addition of *L. monocytogenes* (cheeses BDF and ACE, respectively), were ripened at 10°C for 90 days. Diversity analysis determined that the microbiomes of these two cheese types were significantly different, indicating that the presence of *L. monocytogenes* may alter the microbial community of Gouda cheese. Overall, the relative abundances of the major identified taxa in both types of cheeses trended similarly during ripening. Starter culture bacteria, along with non-subspecies classified *L. lactis*, decreased in abundance during ripening, yet remained the dominant taxa throughout the 90-day period. Other bacterial taxa identified during ripening with relative abundances >1% included *B. thuringiensis*, *Lb. curvatus*, *S. aureus*, and *Str. pyogenes*. These genera have previously been isolated from Danbo, Stilton, and Gouda cheeses (Antonsson et al., 2003; Ercolini et al., 2003; Hajikhani et al., 2007; Salazar et al., 2018) and have either originated from the milk used to make the cheese (as with this study) or from post-process contamination. *S. aureus* and *Str. pyogenes*, which were identified in both types of cheeses at relative abundances >3.60% after 90-day ripening, are both human foodborne pathogens. Although both of these strains were identified in the unpasteurized milk used to make the Gouda cheese in this study, this finding also highlights the need for proper cheese ripening conditions and personnel hygiene.

Although the abundances of the dominant bacterial community members of Gouda cheeses ACE and BDF were similar during ripening, notable differences in the relative abundances of non-dominant taxa were observed. With relative abundances of 0.1–0.5%, a total of 47 and 26 taxa were identified in cheeses ACE and BDF after 90-day ripening, respectively. Interestingly, no taxa were unique to cheese BDF since all the 26 taxa identified at an abundance of 0.1–0.5% in cheese BDF were also identified in cheese ACE. Twenty-one taxa (species from 11 different genera) having relative abundances 0.1–0.5% were unique to cheese ACE after 90 days (see Table 3). The unique taxa included, for example, five species in the genus *Bacteroides*, one species in each of the genera *Flavobacterium*, *Macrococcus*, *Photobacterium*, and *Porphyromonas*, and six species in the genus *Prevotella*.

Within a food matrix, there is a complex interaction between the microbial communities. The addition of a foodborne pathogen, in this case *L. monocytogenes*, may promote growth or hinder the survival of other community members. In contrast, the community members may promote or hinder the survival of *L. monocytogenes*. It is known that certain bacterial taxa, including *Bacillus* spp., *Chryseobacterium* spp., *Enterococcus* spp., *Lactococcus* spp., and *P. fluorescence*, possess anti-listerial properties or can readily out-complete *L. monocytogenes* for nutrients (Carpentier and Chassaing, 2004; Saubusse et al., 2007; Renye et al., 2009). On the other hand, *L. monocytogenes* possesses a bacteriocin, which can target *Prevotella* (Rolhion et al., 2019). In a study assessing the bacterial communities of floor drains in a food production facility both with and without resident *Listeria* spp., *Prevotella* populations were highest in these communities in the absence of *Listeria* spp. (Fox et al., 2014). The lack of identification of certain taxa in cheese BDF after 90 days may be due to inhibition of these bacteria by *L. monocytogenes* or by other community members.

Optimization of *L. monocytogenes* enrichment techniques for a particular food product is often necessary due to co-enriching and competitor resident microbiota. Rapid and accurate enrichment of *L. monocytogenes* from food matrices is essential to outbreak and recall investigations and public health. Some of the taxa present in relative abundances >0.1% in the Gouda cheese after 90 day ripening, regardless of *L. monocytogenes* addition, included *Bacillus*, *Ketobacter*, eight species of *Lactobacillus*, two species of *Pseudomonas*, *Streptomyces*, and *Staphylococcus*. *Bacillus*, *Lactobacillus*, *Pseudomonas*, and *Staphylococcus* species are known to co-enrich and, at times, hinder *L. monocytogenes* detection when cultured in typical *Listeria* enrichment broths (Dallas et al., 1991; Millet et al., 2006). The identification of the bacteria present in Gouda cheese during the ripening process can be used to develop appropriate enrichment procedures for *L. monocytogenes* for this food product.

In conclusion, this study examined the dynamics of the bacterial community in Gouda cheese after manufacture and 90-day ripening when made with unpasteurized milk both with and without the addition of *L. monocytogenes*. Although no microbial community differences were observed in the three batches of unpasteurized milk used to make Gouda cheese in this study, there were significant differences between the microbiomes of these two types of cheeses during ripening. While each type of cheese shared community members, a large number of bacterial taxa were unique to cheese ACE (made without *L. monocytogenes*) after 90-day ripening, indicating that *L. monocytogenes* may influence the microbial community. These data are important to understanding the impact of *L. monocytogenes* on the microbiome of Gouda cheese and vice versa. It is noted that these results are specific to the unpasteurized milk sourced in this study and that future studies could examine metataxonomic changes in Gouda cheese based on the seasonality, farm and cattle, and geographic location of the milk utilized.

## Data Availability Statement

The datasets presented in this study can be found in online repositories. The names of the repository/repositories and accession number(s) can be found in the article/supplementary material.

## Author Contributions

JS, KS, and MT conceived and designed the experiments. JS, LG, and MF performed the experiments and wrote the manuscript. JS, MF, and PR analyzed the data. All authors contributed to the article and approved the submitted version.

### Conflict of Interest

The authors declare that the research was conducted in the absence of any commercial or financial relationships that could be construed as a potential conflict of interest.
